# Screening, detection and management of delirium in the emergency department – a pilot study on the feasibility of a new algorithm for use in older emergency department patients: the modified Confusion Assessment Method for the Emergency Department (mCAM-ED)

**DOI:** 10.1186/1757-7241-22-19

**Published:** 2014-03-13

**Authors:** Florian F Grossmann, Wolfgang Hasemann, Andreas Graber, Roland Bingisser, Reto W Kressig, Christian H Nickel

**Affiliations:** 1 Emergency Department, University Hospital Basel, Basel, Switzerland; 2 Department for Practice Development, University Hospital Basel, Basel, Switzerland; 3 Felix Platter Hospital Basel, University Center for Medicine of Aging, Basel, Switzerland

**Keywords:** Emergency medicine, Emergency nursing, Emergency department, Delirium, Geriatrics, Feasibility, Confusion assessment method

## Abstract

**Background:**

Delirium in emergency department (ED) patients occurs frequently and often remains unrecognized. Most instruments for delirium detection are complex and therefore unfeasible for the ED. The aims of this pilot study were first, to confirm our hypothesis that there is an unmet need for formal delirium assessment by comparing informal delirium ratings of ED staff with formal delirium assessments performed by trained research assistants. Second, to test the feasibility of an algorithm for delirium screening, detection and management, which includes the newly developed modified Confusion Assessment Method for the Emergency Department (mCAM-ED) at the ED bedside. Third, to test interrater reliability of the mCAM-ED.

**Methods:**

This was a pilot study with a pre-post-test design with two data collection periods before and after the implementation of the algorithm. Consecutive ED patients aged 65 years and older were screened and assessed in the ED of a tertiary care center by trained research assistants. The delirium detection rate of informal ratings by nurses and physicians was compared with the standardized mCAM-ED assessment performed by the research assistants. To show the feasibility at the ED bedside, defined as adherence of ED staff to the algorithm, only post-test data were used. Additionally, the ED nurses’ assessments were analyzed qualitatively. To investigate the agreement between research assistants and the reference standard, the two data sets were combined.

**Results:**

In total, 207 patients were included in this study. We found that informal delirium assessment was inappropriate, even after a teaching intervention: Sensitivity of nurses to detect delirium without formal assessment was 0.27 pretest and 0.40 post-test, whilst sensitivity of physicians’ informal rating was 0.45 pre-test and 0.6 post-test. ED staff demonstrated high adherence to the algorithm (76.5%). Research assistants assessing delirium with the mCAM-ED demonstrated a high agreement compared to the reference standard (kappa = 0.729).

**Conclusions:**

Informal assessment of delirium is inadequate. The mCAM-ED proved to be useful at the ED bedside. Performance criteria need to be tested in further studies. The mCAM-ED may contribute to early identification of delirious ED patients.

## Background

Delirium, characterized by acute changes in cognitive status, particularly attention and executive function [1], is common in older ED patients and makes them prone to adverse outcomes such as impaired functional status [2], prolonged hospital stay [3], cognitive decline [4], and increased mortality [5]. Delirium prevalence in older ED patients has been estimated to be between 7% and 10% [6,7].

However, the sensitivity of delirium detection in the ED appears to be poor [8-11]. Possible reasons for low detection rates occur on different levels. Patient-related risk factors such as hypoactive delirium have been identified [12]. Further, mental status screening tools are complex, they are rarely used, and staff is inadequately trained in applying them [13,14]. Environmental factors such as ED crowding, rapid workflow, and high decision density may also contribute. It therefore seems that the time needed to conduct an assessment is first and foremost pivotal for its application [15].

Although several tools for the detection of delirium have been developed, few have been studied in the ED. To our knowledge to date no study has evaluated the utility of formal delirium assessment in the ED setting, using a standardized instrument, compared to informal, clinical delirium detection.

The Confusion Assessment Method (CAM) is a widely used and validated tool for diagnosing delirium [16]. When used by untrained clinicians, however, the sensitivity of the CAM is low [12]. The CAM was validated using the Mini Mental State Examination (MMSE) [17] as a structured patient interview, which is an integral part of the assessment. However, the MMSE is too time-consuming for routine use in the ED, particularly because manual tasks such as writing and drawing are required.

The CAM-ICU is an adaptation of the CAM for use in the intensive care unit [18]. It has also been used for research purposes in the ED [19]. Although the CAM-ICU algorithm can be rapidly performed, the scale was developed for non-verbal responses [20] and may therefore, in addition to its low sensitivity [21], be not ideal for routine use in the ED setting.

Since established delirium screening tools appear to be too complex and too time consuming for the ED setting, or do not provide sufficient information about cognition, there is a need for a quick and sensitive ED screening method. To this end we developed an algorithm for delirium screening, detection and management in older ED patients. An integral part of the algorithm is the modified Confusion Assessment Method for the Emergency Department (mCAM-ED), a feasible approach based on the original short version of the CAM. Recently, Han and colleagues [22] proposed an approach for diagnosing delirium in the ED which is also an adaptation of the CAM. As in our study, the emphasis was on brevity to enhance feasibility. However, delirium assessment was performed by research personnel, and therefore the instrument’s feasibility for ED nurses at the bedside in a busy ED was not shown.

The aims of this study were threefold. First, to investigate whether there is a need for a standardized delirium screening and assessment instrument in the ED. Second, as we hypothesized that such an algorithm would improve delirium detection, we aimed to evaluate the feasibility of our new algorithm for delirium screening, detection and management, which includes the newly developed mCAM-ED as a screening and assessment instrument at the ED bedside. Third, we aimed to assess interrater reliability of the newly developed mCAM-ED.

## Methods

### Study design

For the first aim, a pre-post-test design with two data collection periods was chosen. For our second aim we used only data of the post-test period. This part of the study has a cross sectional design. For the third aim we combined pre- and post-test data sets.

### Study setting and population

The Emergency Department of the University Hospital Basel, Switzerland, has an annual census of about 46,000 visits per year. All patients aged 16 years or older of all specialties except ophthalmology, gynecology and obstetrics, are treated in the ED. There are three possible disposition paths for ED patients: discharge home (or to previous nursing facility), inpatient admission or transfer to another hospital.

The study population consisted of a sample of consecutive ED patients. Patients had to be aged 65 or older to be eligible. The cutoff for age was chosen based on the WHO definition of the older or elderly person [23]. We excluded patients treated in the resuscitation room, those who were transferred or discharged within 2 hours of arrival, as well as patients with insufficient proficiency in the German language or an inability to communicate (e.g. aphasic patients, patients with coma).

### The mCAM-ED

The mCAM-ED is based on the original CAM algorithm developed by Inouye [16] and was adapted with permission. In a first step, ED patients aged 65 years or older are screened for inattention by ED nurses, similar to the CAM-ICU approach [18]. Unlike the ICU, most ED patients are able to perform verbal tasks, so we used a timed recitation of the months of the year in reverse order from the Bedside Confusion Scale (BCS) [24] to assess inattention. Every omission scored one point. A delay of greater than 30 seconds scored one additional point. Following the approach of Stillman et al. [24], inattention was present with a score of 3 or more. When the BCS was validated against the CAM to detect delirium, a score of 3 or more had a sensitivity of 94% and a specificity of 85% [24].

According to our algorithm, the CAM assessment was completed only in case of inattention. To minimize screening burden due to manual tasks such as drawings, and to increase feasibility, we used the Mental Status Questionnaire (MSQ) [25] as a structured interview instead of the MMSE. The MSQ is a 10-item screener for cognitive impairment with a sensitivity of 55% to 70% and a specificity of 96% to 98% for the detection of dementia [26,27]. No manual tasks are necessary to complete the MSQ, which makes it easier and faster than the MMSE. In addition, no licensing fees are charged for its use. Disorganized thinking was present when “disorganized or incoherent speech, such as rambling or irrelevant conversation, unclear or illogical flow of ideas” [28] occurred during the interview or when the Comprehension Test scored less than three. The Comprehension Test by Hart et al. [29] was originally designed to assess the presence of delirium in ICU patients and is a subscale of the Cognitive Test for Delirium (CTD). Questions such as “Will a stone float on water?” and “Can you use a hammer to pound nails?” challenge logical reasoning. Inouye et al. developed the original CAM algorithm in 1990 in a four- and ten-item-version. In both versions, acute onset and a fluctuating course of cognitive alteration, as well as inattention and disorganized thinking or altered consciousness are required to diagnose delirium. For the mCAM-ED, the short version of the CAM and the “or”-criterion is used, i.e. acute onset *or* fluctuating course, to improve sensitivity [28]. With this modification the mCAM-ED requires a maximum of one minute to rate attention and 3 to 5 minutes to complete the in-depth assessment using the structured interview.

### Study protocol

**Figure 1:**
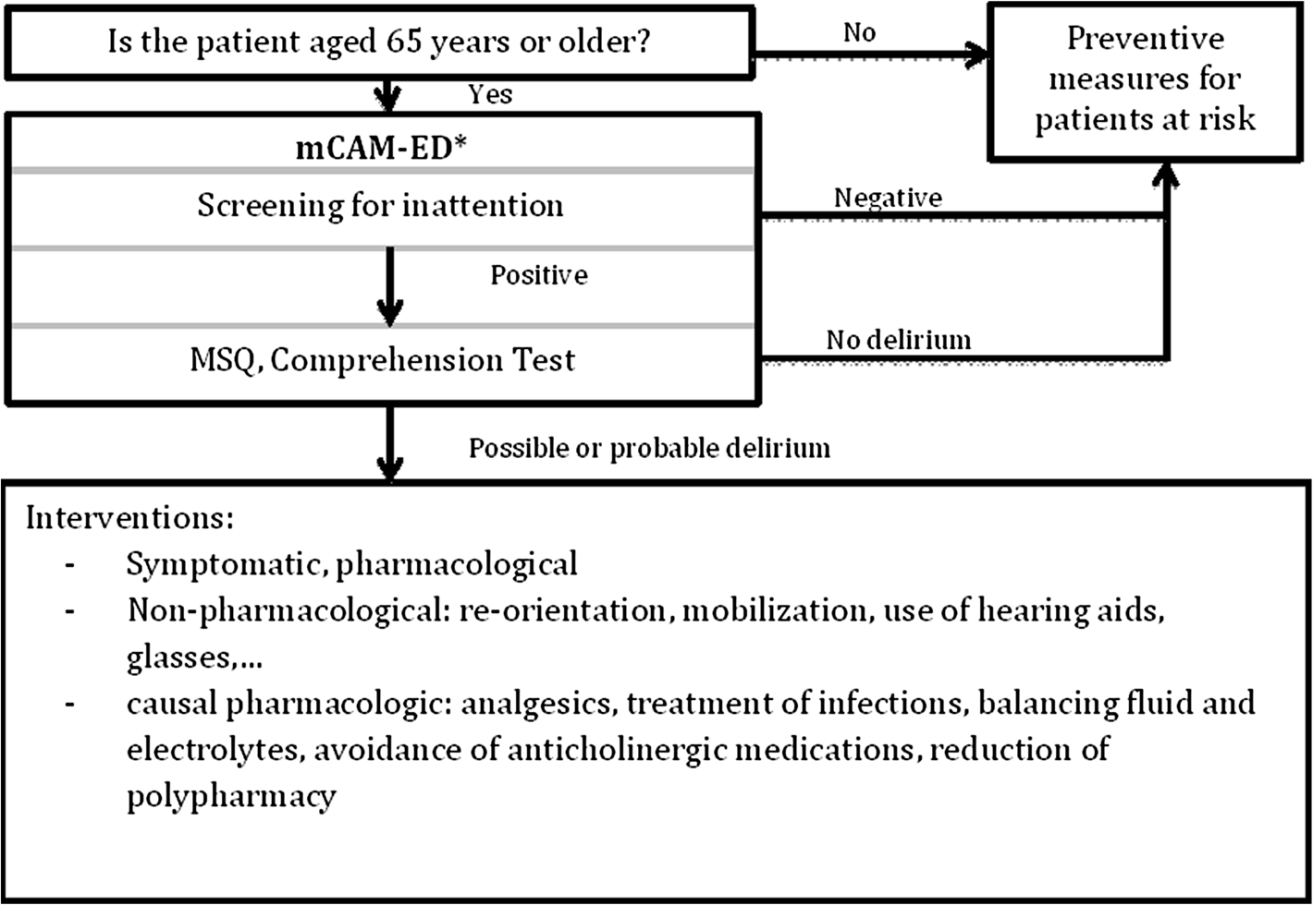
The new algorithm for delirium detection and management in older ED patients including the mCAM-ED.* Interpretation: Inattention plus acute onset or fluctuating course of the cognitive alterations plus disorganized thinking or altered consciousness: possible or probable delirium, every other finding: no delirium. CAM Algorithm adapted from: Inouye SK, et al. Ann Intern Med. 1990; 113: 941–948 [16]. Confusion Assessment Method. Copyright 2003, Hospital Elder Life Program, LLC. Not to be reproduced without permission.

Baseline data were collected in the pre-test period between August 22nd and August 28th 2011. All ED patients who presented to the ED and who met the inclusion criteria were prospectively screened and assessed for delirium by research assistants with the mCAM-ED. Between September 2011 and December 2011 all ED nursing staff was trained in applying the algorithm for screening, detection and management of delirium in a four hour teaching session. Physicians received two half-hour lectures about delirium diagnosis and management. Both ED physicians and ED nurses discussed individual video case presentations. In January 2012, the algorithm for detection and management of delirium in older ED patients, which includes the mCAM-ED, was implemented (see Figure 1). It consists of three elements:

1. Screening: The ED bedside nurses in charge screen for inattention in all patients aged 65 or older before admission to an inpatient ward.

2. Assessment: In case of inattention, an in-depth delirium assessment with the mCAM-ED is completed by the ED nurses.

3. Prevention and management of delirium: Non-pharmacological preventive strategies were offered to prevent delirium in high risk patients. In patients with confirmed delirium, diagnostic procedures, causal and symptomatic treatment, as well as non-pharmacological interventions were conducted.

The use of the algorithm is mandatory for all ED nurses and must be applied to all patients aged 65 or older who are admitted to an inpatient bed.

During the post-test period (between August 13th and August 19th of 2012), trained research assistants screened and assessed all included patients for presence of delirium using the mCAM-ED in addition to the ED nurses at the bedside. By comparing the assessment results, the ED nurses’ performance was investigated. Patients who were identified as having delirium by the research assistants were presented to a senior emergency physician, who formally applied the mCAM-ED to verify the assessment result. The senior emergency physician (CHN) served as the reference standard. The final delirium diagnosis was based on the DSM IV criteria [1].

### Measurements

*Demographic data* included age, sex, Emergency Severity Index level [30], type of presenting complaint according to a published framework [31], number of different medications taken per patient, exposure to anticholinergic drugs according to the anticholinergic cognitive burden scale [32], age adjusted Charlson Comorbidity Index [33].

*Informal delirium detection*: ED nurses and ED physicians in charge were interviewed by the research assistants as to whether they rated a patient - without performing a formal assessment - as having delirium or not. ED nurses and ED physicians were blinded to the research assistants’ mCAM-ED result.

*Delirium* was assessed using the newly developed mCAM-ED. A patient was defined as having delirium if the research assistants diagnosed delirium using the mCAM-ED and if the diagnosis was verified by the senior emergency physician (CHN), who was part of the study group. In case of non-correspondence of the assessments the physician’s diagnosis was the definitive one. In cases where the physician was not available, the research assistants’ diagnosis was kept as the final diagnosis.

*Feasibility* was determined by applying two surrogates, first, the adherence to the algorithm, and second, the nurses’ ability to correctly perform the mCAM-ED.

1. We defined adherence to the algorithm for delirium detection and management as achieved when the “screening and assessment sheet” was filled in by the ED nurse. The mandatory documentation sheet for patient’s valuables served as control variable. This form must be filled in either by ED nurses or by ED nurse assistants and its completion is controlled by the head nurse in charge before the patient is transferred to the ward. The only exceptions are patients who are rapidly transferred to an ICU. In addition to assess adherence to the management part of the algorithm, we reviewed the charts of patients with delirium to ascertain whether delirium was acknowledged in the patient’s problem list of the medical documentation as well as to check whether adequate pharmacological interventions were offered.

2. The ability of the ED nurses to correctly perform the mCAM-ED was assessed qualitatively by reviewing the mCAM-ED sheets filled out by the nurses at the bedside. This was performed by two investigators (FFG and WH) who reviewed the assessment forms.

### Data collection

Patients in both the pre- and post-test periods were formally assessed for delirium by specially trained research assistants. All research assistants were registered nurses with at least a bachelor’s degree, comparable qualification or higher degrees such as a master’s degree. They participated in a 6 day training course, consisting of one eight-hour theory training session, including video examples to learn the mCAM-ED algorithm, followed by 5 days of practical bedside training, provided by an expert in the care of delirious patients (WH). During data collection, the research assistants were supervised by two experienced delirium experts (WH and MS).

In the pre-test period the research assistants assessed consecutive patients over a period of one week between 7 am and 5 pm. During the post-test period, assessment occurred consecutively during 24 hours over a period of one week. Patients were assessed two hours after arrival in the ED at the earliest, and four hours after arrival at the latest. Additionally, in the post-test period, the ED nurses’ screening and assessment results, and information on delirium management were collected from the patients’ charts by two chart abstractors (WH and FFG).

### Statistical analysis

Demographic data and prevalence of delirium were analyzed descriptively. For the informal, clinical delirium detection, sensitivity and specificity were calculated. Differences in sensitivity and specificity between the pre- and post-test periods were calculated by bootstrapping using 95% confidence interval and corresponding p-value. Adherence in the post-test data set was analyzed as the percentage of cases in which the ED nurses performed a screening for inattention where a screening was mandatory. The comparison of the ED nurses’ mCAM-ED results with the results of the research assistants was analyzed descriptively. Agreement between research assistants and the senior emergency physician was analyzed in the data set combining pre- and post-test periods with Cohens’ kappa using bootstrap 95% confidence intervals.

Throughout all analyses, significance was set at a level of 5%. Analyses were performed with SPSS and R. This study was approved by the local ethics committee in charge (identifier EKBB 238/04), and is registered with ClinicalTrials.gov (identifier NCT02054169).

## Results

In total, 340 patients aged 65 and older presented to the ED during the data collection periods. Reasons for exclusion were rapid discharge or transfer to other care facilities (78 patients), instability (29 patients), communication barrier (21 patients), and refusal (5 patients). For the final analysis, 207 patients could be included.

In the pre-test period 74 patients were included and 133 in the post-test period. Demographic characteristics are shown in Table 1. For the outcome variable delirium we detected a significant difference in prevalence rates between the pre-test (16.4%) and the post-test period (5.5%) (p = 0.021). In total, the prevalence rate of delirium in our ED was 9.5%.

**Table 1:** Sample characteristics (n = 128), and prevalence of delirium

	**Pre-test**	**Post-test**	P value*
	**(n = 74)**	**(n = 133)**	
Female sex (n = 207)	48 (64.9%)	70 (52.6%)	0.119
Median age (IQR) in years (n = 207)	80.0 (74.0, 84.8)	76.9 (72.3, 84.2)	0.282
Emergency severity index level (n = 207)			0.493
ESI 1	1 (1.4%)	1 (0.8%)	
ESI 2	21 (28.4%)	28 (21.1%)	
ESI 3	34 (45.9%)	66 (49.6%)	
ESI 4	5 (6.8%)	17 (12.8%)	
ESI 5	0	0	
Missing (direct boarders)	13 (17.6%)	21 (15.8%)	
Complaint (n = 206)			0.427
Specific	40 (54.1%)	81 (61.4%)	
Non specific	17 (23.0%)	21 (15.9%)	
Trauma	12 (16.2%)	25 (18.9%)	
Other	5 (6.76%)	5 (3.79%)	
Disposition			0.113
Admitted	42 (65.8)	85 (63.9%)	
Transferred	10 (13.5%)	7 (5.3%)	
Discharged	22 (29.7%)	41 (30.8%)	
Time between presentation and assessment in hours (n = 207), mean (SD)	2.1 (0.6)	1.9 (0.6)	0.014
Number of different medications (n = 202) mean (SD)	7.58 (5.75)	7.83 (6.85)	0.779
Sum of ACB-score (n = 202)			0.948
0	42 (57.5%)	75 (58.1%)	
1	19 (26.0%)	35 (27.1%)	
2	5 (6.85%)	10 (7.7%)	
≥3	7 (9.59%)	9 (7.0%)	
Age adjusted CCI (n = 203), median (IQR)	5.0 (4.0, 7.0)	5.0 (4.0, 6.0)	0.229
Delirium, yes (n = 201)			0.021
Yes	12 (16.4%)	7 (5.5%)	
No	61 (83.6%)	121 (94.5%)	

ESI, Emergency Severity Index; ACB, Anticholinergic Cognitive Burden Scale; CCI, Charlson Comorbidity Index, SD, standard deviation.

*T-Test, Mann–Whitney U test and Chi-square test as appropriate.

When rating the presence of delirium in patients without formal assessment, both nurses and physicians performed weakly. The sensitivity and specificity estimates are shown in Tables 2 and 3. Differences between the pre-test and post-test periods were not significant, except for the specificity of the nurses’ informal ratings (p = 0.012).

**Table 2:** Performance of nurses’ informal delirium ratings pre and post-test*

**Pre test**				**Post-test**			
	**Gold standard°**				**Gold standard°**		
**Nurses’ ratings**	**Delirium**	**No Delirium**	**Total**	**Nurses’ ratings**	**Delirium**	**No delirium**	**Total**
Delirium	3	0	3	Delirium	2	7	9
No delirium	8	59	67	No delirium	3	108	111
Total	11	59	70	Total	5	115	120
Sensitivity: 0.27				Sensitivity: 0.40			
Specificity: 0.98				Specificity: 0.94			

*In the post-test period ED nurses were interviewed to informally rate prevalence of delirium before they conducted a formal mCAM-ED assessment.

°The research assistants’ mCAM-ED ratings, confirmed by the senior emergency physician’s re-assessment served as gold standard.

**Table 3:** Performance of physicians’ informal delirium ratings pre and post-test

**Pre-test**				**Post-test**			
	Gold standard*				Gold standard*		
**Physicians’ ratings**	**Delirium**	**No delirium**	**Total**	**Physicians’ ratings**	**Delirium**	**No delirium**	**Total**
Delirium	5	2	7	Delirium	3	7	10
No delirium	6	56	62	No delirium	2	107	109
Total	11	58	69	Total	5	114	119
Sensitivity: 0.45				Sensitivity: 0.6			
Specificity: 0.97				Specificity: 0.94			

*The research assistants’ ratings with the mCAM-ED, confirmed by the senior emergency physician’s re-assessment served as gold standard.

Of 85 patients who were admitted to an inpatient ward, 65 were screened and, if indicated, an assessment performed by the ED nurses. This corresponds to an adherence rate of 76.5%. An additional 9 patients were screened by ED nurses where this was not mandatory: 7 patients who were later discharged and 2 patients who were transferred. Non-adherence occurred in 20 cases where no screening was performed despite being indicated. In this group there were two patients with delirium, diagnosed by the research assistants. Adherence to the documentation sheet for patient’s valuables was 75.6% for registered ED nurses and 97.6%, when nurse assistants were included into the analysis. Delirium management was analyzed in the 7 patients where delirium was identified (Table 4). In 5 cases adequate medication was prescribed or administered, respectively. In 5 cases delirium was mentioned in the problem list. These two groups only partially overlap. There was one case where neither delirium was mentioned in the problem list nor was adequate medication offered.

**Table 4:** Management of delirium

**No.**	**mCAM-ED (Assessment by the ED nurses)**	**Disposition**	**Management**	
			**Delirium documented in problem-list**	**Adequate medication**
1	Delirium	Admitted	Yes	Yes
2	Missing	Transferred^a^	No	No
3	No delirium	Admitted	No	Yes
4	No delirium	Admitted	Yes	Yes
5	Missing	Admitted	Yes	Yes
6	Delirium	Transferred^a^	Yes	No
7	Missing	Discharged^a,b^	Yes	Yes

^a^In patients who were transferred or discharged, a screening /assessment was not mandatory for the ED nurses.

^b^This patient was discharged home against advice on the family’s request.

When analyzing the ED nurses’ ability in correctly applying the mCAM-ED, 74 patients’ “screening and assessment sheets”, could be included. 65 screenings resulted in normal attention, and 9 patients were positive for inattention. When all 74 ED nurses’ “screening and assessment sheets” were analyzed qualitatively, we detected 6 assessments (8.1%) that were not conclusive. In one case, no screening was documented, but parts of the assessment were completed. In one other case the assessment was omitted despite the presence of inattention. In four cases results of the formal assessment instruments were inadequately interpreted. Among these 6 cases no patient was diagnosed as having delirium by the research assistants.

Out of the 7 patients with delirium, four patients were admitted. In those a screening/assessment would have been mandatory. Of those four patients, one was correctly identified by ED nurses as having delirium, two patients were not identified as delirious, and one patient had no screening/assessment. The small number of delirious patients in this sample did not allow calculation of psychometric properties.

Agreement between research assistants and the senior emergency physician resulted in a Cohen’s kappa of 0.729 (95% CI 0.36-1.00). They agreed in 86.6% of the cases (13 out of 15 cases, 95% CI 0.595-0.983).

## Discussion

In this pilot study we showed informal, clinical delirium rating to be inadequate, even after a teaching intervention, confirming the need for a standardized, formal delirium screening and assessment instrument in the ED. The feasibility of the new algorithm for delirium screening, detection, and management in older ED patients, which includes the newly developed mCAM-ED, is suggested by first, high adherence to the algorithm, and second by the promising results of the ED nurse’s ability in correctly applying the mCAM-ED. Due to small sample sizes in this pilot study we were not able to calculate sensitivity and specificity. Agreement between research assistants and the emergency physician reference standard is high, suggesting the validity of the mCAM-ED.

Few other studies have investigated tools for delirium detection in the ED. Fabbri et al. [34] validated the Brazilian version of the Confusion Assessment (CAM) in an ED setting more than 20 years ago, achieving a sensitivity of 94.1% and a specificity of 96.4%, when ratings of ED physicians were compared with geriatricians’ ratings using DSM-IV criteria. Despite these promising results, important information, such as how the structured interview was conducted, is missing. Monette et al. [35] compared nurses’ ratings of the CAM with those of geriatricians in the ED. After 5 days of training the nurses’ ratings achieved a sensitivity of 86% and specificity of 100%. Han and colleagues [22] showed that research assistants, similarly trained to those in our study, achieved high sensitivity and specificity in diagnosing delirium versus a psychiatrist reference standard. The finding that trained research assistants can reliably perform a delirium assessment is in line with our study. However, apart from brevity, Han et al. [22] could not draw any conclusions regarding the instrument’s feasibility for ED nurses at the bedside in a busy ED.

Delirium often remains unrecognized in the hospital course when missed in the ED [7]. For this reason, an instrument targeted to the ED setting is necessary. Our mCAM-ED algorithm proved to be feasible at the ED bedside. This is shown by first the high adherence rate which is comparable to a control surrogate (the patients’ valuables forms which are mandatory for admitted patients). Secondly, we found a low rate of inappropriately filled screening and assessment sheets. In addition, the initial screening for inattention can be performed rapidly. Therefore, this approach is ideal for a busy ED setting with a high patient turnover and high workload. By these means, the burden of screening is reduced to a minimum for the patient as well as for ED staff.

However, with regard to the assessment, two patients were rated as non-delirious although research assistants diagnosed delirium. Perhaps, an IT-based assessment form calculating the final score might solve this problem, as nurses reported that they did not find the layout of the assessment part of the “screening and assessment sheet” to be straight-forward. Another option is to train ED nurses in delirium screening only and have specially trained providers complete the assessment with the mCAM-ED.

As our study was designed as a pilot study, we suggest measuring diagnostic performance and validating the new algorithm in a larger patient sample in different settings. Furthermore, the impact of our algorithm on patient outcomes should be investigated.

### Limitations

This was a pilot study with a small sample size. The pre and the post-test samples differed in prevalence of delirium. This may be due to increased awareness and better prevention after the implementation of the algorithm. It may also be a consequence of different patient populations. However, baseline characteristics suggest otherwise. Due to the low prevalence of delirium in a small sample, sensitivity and specificity of the mCAM-ED could not be calculated. However, a strength of our study is that all patients included in our study were screened for inattention following our approach (consecutive sampling procedure).

Further, formal validation of the instrument was not subject of this study. A senior emergency physician was used as a reference standard. In five cases in which the research assistants diagnosed delirium, the senior emergency physicians’ rating was not available. However, high agreement between the research assistants and reference standard in our and in other studies [22] may attenuate this effect. Further, only patients with a positive delirium assessment result by the mCAM-ED were evaluated by the reference standard. This may have led to omission of false negatives.

Whether the four-hour training of the ED nurses was sufficient, cannot be answered with this study. However, our data suggest that a high interrater reliability can be achieved after a 6 day training period, comparing ratings of the research assistants with the reference standard. Similar results were found by Monette et al. [35] and Han et al. [22].

## Conclusions

Informal, clinical delirium rating is inadequate. However, delirium screening, detection, and management with our algorithm, which includes the newly developed mCAM-ED is feasible at the ED bedside. It may contribute to safe emergency care for the vulnerable group of older ED patients.

## Competing interests

The authors declare that they have no competing interests.

## Authors’ contributions

FFG, WH and CHN conceived the study and designed the trial. AG, WH, FFG, and CHN collected and analyzed the data. All authors contributed to the interpretation of the findings. FFG and CHN drafted the manuscript and all authors contributed to its revision. All authors read and approved the final manuscript.
